# Gangrenous Cecal Volvulus Complicating Puerperium: Is the Delay in Diagnosis Really Inevitable?

**DOI:** 10.1155/2012/236109

**Published:** 2012-05-31

**Authors:** Chanderdeep Sharma, Shashank Shekhar, Satish Kumar, Rajesh Chaudhary

**Affiliations:** ^1^Department of Obstetrics & Gynecology, Dr. RPGMC at Tanda, Set No. 112, Vivekanand Resident Hostel, Tanda 176001, India; ^2^Department of General Surgery, Dr. RPGMC at Tanda, Tanda 176001, India

## Abstract

Volvulus in puerperium is a rare event. We present a case of cecal volvulus following normal vaginal delivery that ended with cecal gangrene and hemicolectomy.

## 1. Introduction

Intestinal obstruction is a very rare event during pregnancy with reported incidence varying widely between 1 in 1500 to 1 in 66431 [1]. The most common cause of intestinal obstruction during pregnancy and puerperium is postsurgical adhesions (60–70%) followed by volvulus, which is seen in 25% of cases [1, 2]. Due to a host of reasons peculiar to pregnancy and puerperium diagnosis is often delayed and thus a simple mechanical obstruction of viable gut can become a serious case of volvulus with gangrene and life-threatening complications. We report a case of cecal volvulus during puerperium with delayed diagnosis leading to bowel gangrene to highlight the consequences and to ask a valid question: is the delay in diagnosis during pregnancy and puerperium really inevitable?

## 2. Case Report

**Figure 1:**
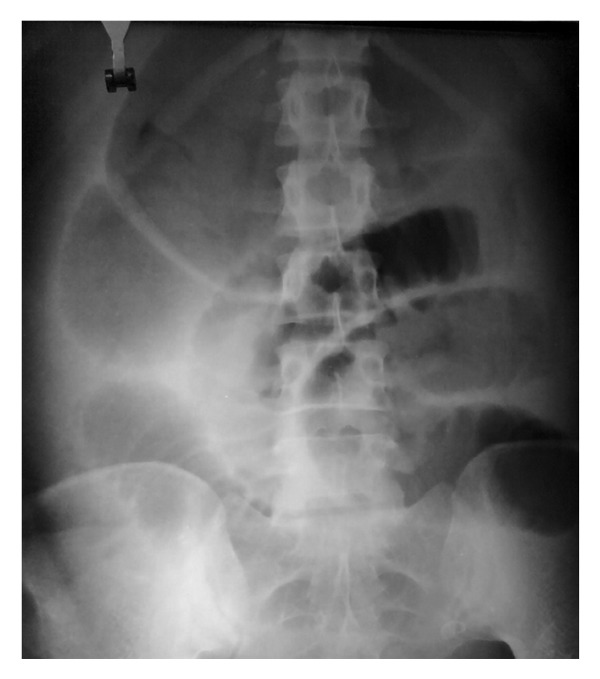
Plain abdominal radiograph with dilated colon.

**Figure 2:**
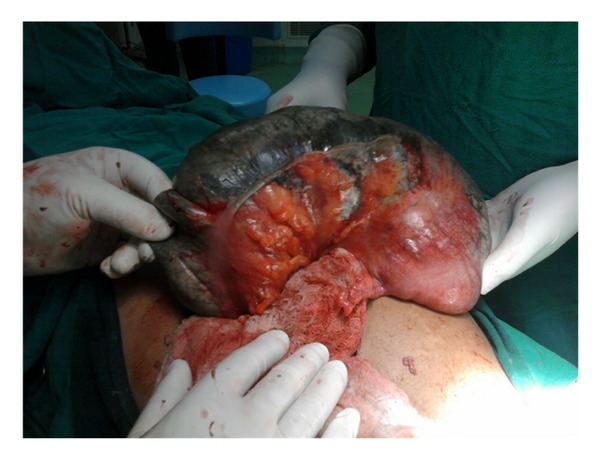
Surgical findings with gangrenous cecal volvulus.

A 26-year-old para two was referred to our hospital on third postpartum day with history of sudden onset lower abdominal cramping pain, abdominal distension, vomiting, and obstipation for two days. The patient had a spontaneous term uncomplicated vaginal delivery at home. The patient was earlier managed at a peripheral health institute conservatively and was referred to our hospital when her condition deteriorated. Her previous delivery was home conducted as well. There was no history of abdominal surgery in the past. Examination revealed moderate pallor and tachycardia. Abdomen was grossly distended and tender. Bowel sounds were absent. Routine investigations revealed hemoglobin of 6.4 gm% and an elevated leucocytic count of 16,000 per cubic mm. Simple abdominal radiograph revealed a hugely distended large bowel segment in the upper quadrant as shown in Figure 1. The general condition of the patient worsened in the next few hours with appearance of signs of peritonism. Leucocytic count soared to 21000 per cubic mm, and decision for immediate laparotomy was taken with a provisional diagnosis of large bowel obstruction. On exploration, there was a grossly distended, gangrenous cecum displaced in the upper left quadrant as shown in Figure 2. The gangrenous gut was resected with ileotransverse anastomosis. The patient had uneventful recovery and was discharged on day 15. 

## 3. Discussion

Volvulus of the cecum is an axial twisting or folding of cecum on its mesentery that results in bowel obstruction [3]. The incidence of volvulus is the greatest at times of rapid changes in uterine size and can occur during mid pregnancy, third trimester, and more commonly during puerperium [4]. The most common site of volvulus in pregnancy is sigmoid colon followed by small bowel and cecum [4, 5]. It has been postulated that uterine enlargement during pregnancy pushes up the redundant or abnormally mobile cecum out of the pelvis, which increases the chances of its axial rotation around a fixed point. During puerperium a different set of events occur; there is rapid reduction of intra-abdominal contents, which may alter the mechanical equilibrium of the bowel and mesenteric base leading to twisting of bowel segments [6]. Volvulus gives rise to twin sets of clinical findings due to two components, namely, mechanical obstruction and vascular compromise. Pregnancy, however, may obscure these signs due to enlarged gravid uterus displacing and shielding the abdominal viscera from clinical scrutiny and symptoms might be mistaken for common discomfort of pregnancy thus delaying the diagnosis. The diagnosis of acute abdomen is also delayed during puerperium, which, however, cannot be explained by large uterus obscuring the signs. Increased abdominal girth and difficulty of eliciting signs of peritonism due to lax abdominal wall have been suggested as explanations for failure to diagnose acute abdomen early in puerperium [7]. X-ray abdomen is highly sensitive (95%) investigation for diagnosis of cecal volvulus. A characteristic “coffee bean” deformity may be seen directed towards the left upper quadrant as seen in our patient, which, however, was appreciated retrospectively. But dilated cecal loop may appear anywhere in the abdomen due to its abnormal mobility [8], and it has been suggested that volvulus should be suspected when there is a single grossly dilated loop of bowel on radiograph [9]. The therapeutic algorithm is the same for pregnant and non pregnant women. Surgical treatment of cecal volvulus consists of untwisting the bowel, decompressing the distended segments, removing the devitalized tissue, and preventing recurrence. The surgical techniques described for cecal volvulus are cecostomy, cecopexy, resection with ileostomy, and resection with primary anastomosis. 

Though a rare event, the incidence of intestinal obstruction during pregnancy and puerperium is on the rise due to increasing rate of cesarean deliveries. Hence, an obstetrician is likely to come across a case of bowel obstruction with pregnancy or puerperium in his or her lifetime. Because of peculiar changes of pregnancy and puerperium the diagnosis is often delayed leading to life-threatening complications. We must ask ourselves: is the delay in diagnosis really inevitable? Cecal gangrene ensues in an average duration of 48–72 hours. A high index of suspicion, urgent X-ray in a patient of acute abdomen, followed by computerized tomography if required and laparotomy within golden time that is, 24 hours of onset may prevent gangrene and resultant perforation of the intestine. We believe that a heightened awareness amongst obstetricians regarding the possibility and knowledge of diagnostic tools and their immediate and liberal use, in particular radiograph, can and should prevent the delay in diagnosis.
